# Middle East Respiratory Syndrome Coronavirus Nonstructural Protein 16 Is Necessary for Interferon Resistance and Viral Pathogenesis

**DOI:** 10.1128/mSphere.00346-17

**Published:** 2017-11-15

**Authors:** Vineet D. Menachery, Lisa E. Gralinski, Hugh D. Mitchell, Kenneth H. Dinnon, Sarah R. Leist, Boyd L. Yount, Rachel L. Graham, Eileen T. McAnarney, Kelly G. Stratton, Adam S. Cockrell, Kari Debbink, Amy C. Sims, Katrina M. Waters, Ralph S. Baric

**Affiliations:** a Department of Microbiology and Immunology, University of Texas Medical Branch, Galveston, Texas, USA; b Department of Epidemiology, University of North Carolina at Chapel Hill, Chapel Hill, North Carolina, USA; c Department of Microbiology and Immunology, University of North Carolina at Chapel Hill, Chapel Hill, North Carolina, USA; d Pacific Northwest National Laboratory, Richland, Washington, USA; e Department of Natural Sciences, Bowie State University, Bowie, Maryland, USA; Icahn School of Medicine at Mount Sinai

**Keywords:** IFIT, MERS-CoV, SARS-CoV, coronavirus, emerging virus, live attenuated, methyltransferase, vaccine

## Abstract

Coronaviruses (CoVs) encode a mixture of highly conserved and novel genes, as well as genetic elements necessary for infection and pathogenesis, raising the possibility of common targets for attenuation and therapeutic design. In this study, we focused on highly conserved nonstructural protein 16 (NSP16), a viral 2′*O*-methyltransferase (2′*O*-MTase) that encodes critical functions in immune modulation and infection. Using reverse genetics, we disrupted a key motif in the conserved KDKE motif of Middle East respiratory syndrome CoV (MERS-CoV) NSP16 (D130A) and evaluated the effect on viral infection and pathogenesis. While the absence of 2′*O*-MTase activity had only a marginal impact on propagation and replication in Vero cells, dNSP16 mutant MERS-CoV demonstrated significant attenuation relative to the control both in primary human airway cell cultures and *in vivo*. Further examination indicated that dNSP16 mutant MERS-CoV had a type I interferon (IFN)-based attenuation and was partially restored in the absence of molecules of IFN-induced proteins with tetratricopeptide repeats. Importantly, the robust attenuation permitted the use of dNSP16 mutant MERS-CoV as a live attenuated vaccine platform protecting from a challenge with a mouse-adapted MERS-CoV strain. These studies demonstrate the importance of the conserved 2′*O*-MTase activity for CoV pathogenesis and highlight NSP16 as a conserved universal target for rapid live attenuated vaccine design in an expanding CoV outbreak setting.

**IMPORTANCE** Coronavirus (CoV) emergence in both humans and livestock represents a significant threat to global public health, as evidenced by the sudden emergence of severe acute respiratory syndrome CoV (SARS-CoV), MERS-CoV, porcine epidemic diarrhea virus, and swine delta CoV in the 21st century. These studies describe an approach that effectively targets the highly conserved 2′*O*-MTase activity of CoVs for attenuation. With clear understanding of the IFN/IFIT (IFN-induced proteins with tetratricopeptide repeats)-based mechanism, NSP16 mutants provide a suitable target for a live attenuated vaccine platform, as well as therapeutic development for both current and future emergent CoV strains. Importantly, other approaches targeting other conserved pan-CoV functions have not yet proven effective against MERS-CoV, illustrating the broad applicability of targeting viral 2′*O*-MTase function across CoVs.

## Abstract (precis)

Coronavirus (CoV) emergence in both humans and livestock represents a significant threat to global public health, as evidenced by the sudden emergence of severe acute respiratory syndrome CoV (SARS-CoV), MERS-CoV, porcine epidemic diarrhea virus, and swine delta CoV in the 21st century. These studies describe an approach that effectively targets the highly conserved 2′*O*-MTase activity of CoVs for attenuation. With clear understanding of the IFN/IFIT (IFN-induced proteins with tetratricopeptide repeats)-based mechanism, NSP16 mutants provide a suitable target for a live attenuated vaccine platform, as well as therapeutic development for both current and future emergent CoV strains. Importantly, other approaches targeting other conserved pan-CoV functions have not yet proven effective against MERS-CoV, illustrating the broad applicability of targeting viral 2′*O*-MTase function across CoVs.

## INTRODUCTION

The emergence of Middle East respiratory syndrome coronavirus (MERS-CoV) in 2012 represents the second severe CoV to strike the human population since the beginning of the 21st century (1). Similar to its predecessor, severe acute respiratory syndrome CoV (SARS-CoV), MERS-CoV is characterized by severe lung infection and high mortality rates (2). Associated with elderly patients and nosocomial spread, MERS-CoV is likely harbored in camel populations with periodic reintroductions into humans, followed by periodic nosocomial outbreaks in hospital settings (3). Importantly, with the continued rate of globalization, MERS-CoV remains an ongoing threat for future outbreaks both in and outside the Middle East, as evidenced by the large outbreak in South Korea (4). Together, these factors highlight the importance of examining CoV pathogenesis and developing conserved therapeutic targets for the treatment of current and future emergent strains.

Like all members of the CoV family, MERS-CoV maintains a balance of conserved and novel viral proteins within its genome (5). It is a member of the group 2C CoV family, and a wealth of distinct accessory open reading frames and nonstructural proteins (NSPs) have already been established to have important roles in modulation of the host immune response (6). Similarly, a number of viral proteins highly conserved in structure, replication, and fidelity are also maintained in the CoV backbone (7). Among these, MERS-CoV NSP16 provides a potent target for therapeutic development. A 2′*O*-methyltransferase (2′*O*-MTase), CoV NSP16 has been implicated in the capping of viral RNA and prevention of its recognition by the intracellular sensor MDA5 and antiviral effectors, including members of the IFIT (interferon [IFN]-induced proteins with tetratricopeptide repeats) family (8). Generation of mutants with changes in the NSP16 KDKE active site resulted in IFN-mediated *in vitro* and *in vivo* attenuation of both mouse hepatitis virus (MHV) and SARS-CoV (9, 10). Therefore, an approach targeting MERS-CoV NSP16 might be anticipated to result in attenuation and potentially provide a universal platform for CoV vaccines against future emergent strains.

Using reverse genetics to target residues in the highly conserved active site, we evaluated MERS-CoV infection outcomes in the context of inactive NSP16 (dNSP16). Consistent with previous studies of SARS-CoV (10), the dNSP16 MERS-CoV mutant maintained no significant attenuation in terms of replication or the initial host immune response. However, both primary human airway epithelial (HAE) cells and *in vivo* studies in a MERS-CoV mouse model demonstrated robust attenuation of dNSP16 mutant growth and pathogenesis. Notably, attenuation was both IFN and IFIT1 dependent, providing a clear mechanism for attenuation. Importantly, the dNSP16 mutant also provided robust protection against a lethal MERS-CoV challenge and maintained attenuation in the mouse-adapted backbone. Together, the results illustrate the broad conservation and necessity of NSP16 in CoV pathogenesis and highlight the targeting of this protein as a rapid-response platform for future emergent CoV strains.

## RESULTS

**FIG 1:**
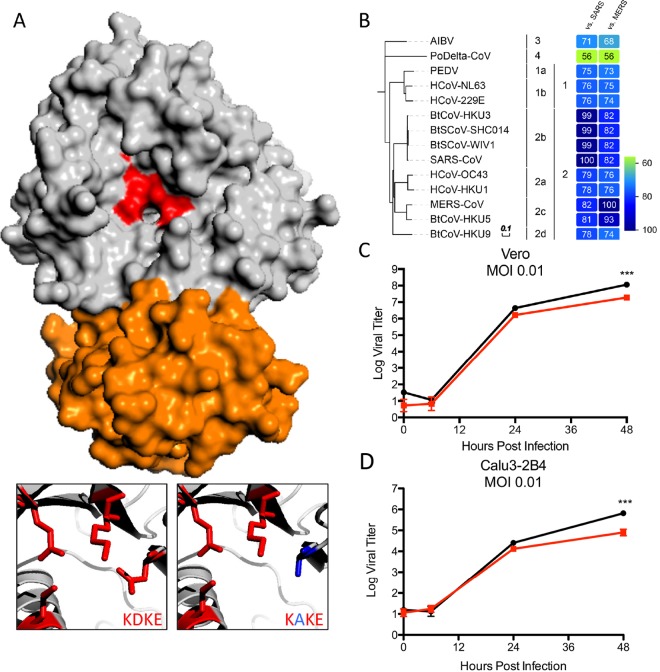
NSP16 is highly conserved in the CoV family. (A) MERS-CoV NSP16-NSP10 complex. Shown is NSP16 (gray) highlighting the conserved KDKE motif (red) required for 2′*O*-MTase activity. Also shown is the NSP10 scaffold required for MERS-CoV (orange). The inset displays conserved KDKE (left) and the D130A mutation (right) that disrupts function. Homology models were created with Modeler in the Max-Planck Institute Bioinformatics Toolkit. The known crystal structure of the NSP16-NSP10 complex (3R24 in the RCSB Protein Data Bank) was used as the template structure (38). Homology models were then manipulated with MacPyMol. (B) Heat maps were constructed from a set of representative CoVs from all four genogroups by using alignment data paired with neighbor-joining phylogenetic trees built in Geneious (v.9.1.5) and visualized in EvolView (http://evolgenius.info/). Trees show the degree of genetic similarity of NSP16 across CoV families. (C and D) Viral replication of dNSP16 mutant MERS-CoV (red) relative to WT MERS-CoV (black) in Vero (C) and Calu-3 2B4 (D) cells following infection at an MOI of 0.01. ***, *P* < 0.001 (Student *t* test).

A combination of structural and biochemical approaches has established a critical role for CoV NSP16 in 2′*O*-MTase activity (Fig. 1A). Stabilized by interactions with NSP10 (orange), NSP16 has been identified as a structurally conserved AdoMet-dependent methyltransferase (11); despite divergence in protein sequence across organisms, an invariant KDKE motif (red) within the methyltransferase core is required to mediate its activity (12). This KDKE motif is highly conserved within all of the NSP16 sequences examined in the CoV family (Fig. 1B). Importantly, mutation of any of the KDKE residues has been shown to ablate 2′*O*-MTase activity (11). In addition, previous alteration of this motif in both group 2b SARS-CoV (10) and group 2a MHV (9) disrupted 2′*O*-MTase activity and attenuated various aspects of infection. On the basis of high conservation in the CoV family, we hypothesized that disruption of the KDKE motif would also attenuate other emerging CoV families, including group 2c MERS-CoV. Utilizing a MERS-CoV reversed genetic system (13), we disrupted the KDKE motif by mutating two nucleotides to produce a D130A change (Fig. 1A). The resulting disrupted-NSP16 (dNSP16) mutant had no significant defect noted in stock titer generation (not shown); similarly, infection of both Vero cells and Calu-3 2B4 cells, a respiratory epithelial cell line, at a low multiplicity of infection (MOI) demonstrated only modest attenuation at late time points (Fig. 1C and D). Together, these results indicate that NSP16 activity is not required for replication.

### Similar *in vitro* host responses of SARS-CoV and MERS-CoV dNSP16 mutants.

**FIG 2:**
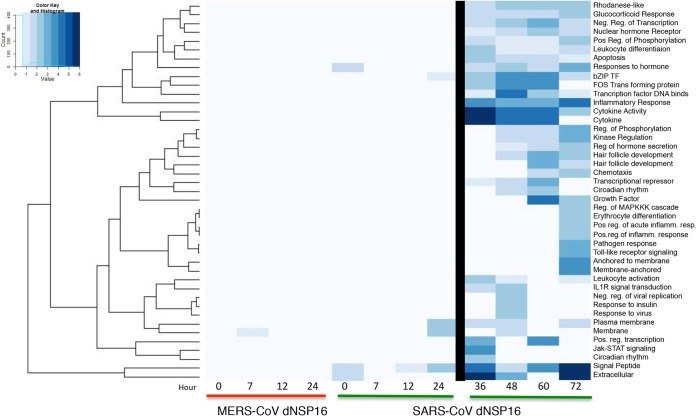
dNSP16 mutant MERS-CoV infection produces minimal changes in early host responses. Changes in functional host gene clusters on the basis of RNA expression following infection at an MOI of 5 of Calu-3 2B4 cells with dNSP16 mutant MERS-CoV (left) or SARS-CoV (right) relative to the WT control virus. The heat map plots significant enrichment of clustered functional categories (as determined by David analysis) for each mutant over time. Only marginal changes were noted during the first 24 h for both SARS-CoV and MERS-CoV dNSP16 mutants. After 24 h (right), significant changes due to SARS-CoV were noted; MERS-CoV had a significant CPE after 24 h postinfection, precluding analysis.

Having established replication competence in both Vero and Calu-3 2B4 cells, we next evaluated the induction of host pathways following infection. Calu-3 2B4 cells infected at an MOI of 5 demonstrated no differences in replication (not shown) and only modest differences in host induction (zero genes with a log_2_ change in expression of >1.5-fold), similar to observations with NSP16 mutant SARS-CoV compared with wild-type (WT) SARS-CoV (10). However, unlike in studies with SARS-CoV, a rapid cytopathic effect (CPE) by 24 h limited the analysis to early time points with WT and dNSP16 mutant MERS-CoV. Further David-based analysis compared the network host responses to the MERS-CoV and SARS-CoV dNSP16 mutants (Fig. 2). Over the first 24 h of infection, both MERS-CoV and SARS-CoV dNSP16 mutant infections showed no significant functional enrichment of any categories relative to corresponding WT infections, consistent with the lack of replication attenuation. However, at late times (>24 h postinfection), SARS-CoV produced robust changes in several host pathways, including cytokine responses, inflammation, and extracellular activity. Similarly, changes in apoptosis, transcription repression, and regulation of phosphorylation indicated a host response more hostile to viral infection. While a more rapid CPE following both WT and dNSP16 mutant MERS-CoV infections precluded an equivalent finding at late time points, the SARS-CoV results suggest that the absence of NSP16 activity eventually initiates host response changes that contribute to attenuation at late time points.

### MERS-CoV dNSP16 mutant attenuated in primary and *in vivo* models.

**FIG 3:**
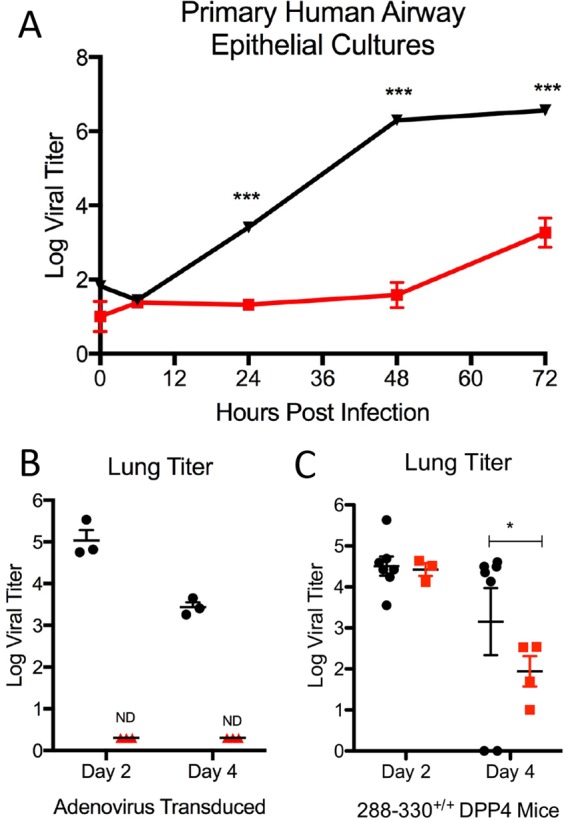
dNSP16 mutant MERS-CoV is attenuated in primary cultures and *in vivo*. (A) Primary HAE cells infected with WT (black) or dNSP16 mutant (red) MERS-CoV at an MOI of 0.01 and monitored over a time course. (B) Day 2 and 4 lung titers of adenovirus-transduced mice expressing human *Dpp4* infected with WT (black) or dNSP16 mutant (red) MERS-CoV. (C) Day 2 and 4 lung titers of 288-330^+/+^ CRISPR-Cas9-targeted mice infected with WT (black) or dNSP16 mutant (red) MERS-CoV. *, *P* < 0.05; **, *P* < 0.01; ***, *P* < 0.001. (Student *t* test). *n* = ≥4 for each experimental group at each time point over multiple experiments.

To further examine the replicative capacity of the dNSP16 mutant, we infected both HAE cells and mice expressing human dipeptidyl peptidase 4, the receptor for MERS-CoV. Primary HAE cell cultures were challenged with WT and dNSP16 MERS-CoV at a low MOI (Fig. 3A). While robust replication was observed following WT infection, dNSP16 MERS-CoV had significant attenuation that corresponded well to previous results obtained with dNSP16 mutant SARS-CoV (10). We next examined dNSP16 mutant MERS-CoV replication phenotypes in the context of *in vivo* infection by using an adenovirus BALB/c mouse transduction model (14). While neither infection produced weight loss (not shown), WT MERS-CoV replicated efficiently at both days 2 and 4 postinfection (Fig. 3B); in contrast, no detectable replication was seen following infection with dNSP16 mutant MERS-CoV. The lack of replication may be due to residual IFN responses associated with initial adenovirus infection. For greater clarity, we next infected CRISPR-Cas9-targeted mice that include mutations in *Dpp4* at positions 288 and 330 (288-330^+/+^) conferring efficient WT MERS-CoV infection and growth in mice but no clinical disease (15). Following infection, no changes were observed in weight loss in either group of mice, consistent with previous findings (data not shown). However, absence of NSP16 activity severely attenuated dNSP16 mutant virus replication at both days 2 and 4 postinfection (Fig. 3C). Coupled with data from HAE cell cultures and the adenovirus model, the results demonstrate clear attenuation of dNSP16 mutant MERS-CoV relative to the control.

### dNSP16 mutant MERS-CoV attenuation is mediated by IFN and IFIT1.

**FIG 4:**
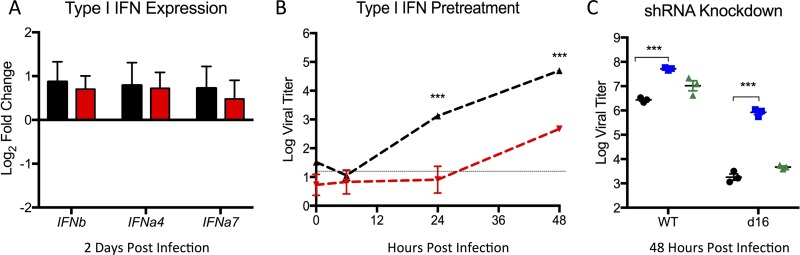
dNSP16 mutant MERS-CoV attenuated by type I IFN treatment via IFIT1. (A) IFN-β, IFN-α4, and IFN-α7 RNA expression in the lungs of 288-330^+/+^ CRISPR-Cas9-targeted mice at 2 days postinfection with WT (black) or dNSP16 mutant (red) MERS-CoV. Values are representative of log_2_ fold expression relative to mock-infected lungs as measured by real-time reverse transcription-PCR. (B) Vero cells were treated with type I IFN (1,000 U) 16 h prior to infection with either WT (black) or dNSP16 mutant (red) MERS-CoV. (C) Vero cells expressing shRNA targeting IFIT1 (blue) or IFIT2 (green) or a no-shRNA control cells (black) were pretreated with IFN-β (PBL Laboratories) and infected with WT (left) or dNSP16 mutant (right) MERS-CoV. *, *P* < 0.05; **, *P* < 0.01; ***, *P* < 0.001 (Student *t* test).

Having established a deficit in dNSP16 mutant MERS-CoV replication in relevant *in vitro* and *in vivo* models, we next sought to evaluate the mechanism of attenuation. Previous work by our lab and others had established increased susceptibility of dNSP16 mutants to type I IFN (9, 10); however, dNSP16 mutant SARS-CoV had not shown augmented type I IFN stimulation following infection (10). Consistent with this finding, infection with dNSP16 mutant MERS-CoV produced stimulation of type I IFN transcript similar to that seen following *in vivo* infection of *Dpp4* mutant (288-330^+/+^) CRISPR mice with the WT virus (Fig. 4A). In contrast, while both the WT and mutant viruses were sensitive to IFN treatment, dNSP16 mutant MERS-CoV had a significant reduction in replication relative to the control virus (Fig. 4B). These attenuation results are consistent with reports of NSP16 mutants of other CoVs and are in contrast to the equivalent replication observed without pretreatment (Fig. 1C) (8). Extending this analysis further, we examined the role of *IFIT1* and *IFIT2* gene expression in this attenuation phenotype in previously constructed stable short hairpin RNA (shRNA) knockdown cell lines (10). Similar to SARS-CoV, knockdown of *IFIT1* augmented replication of dNSP16 mutant MERS-CoV in the context of type I IFN pretreatment (Fig. 4C). In addition, knockdown augmented WT MERS-CoV infection, suggesting sensitivity to *IFIT1* activity despite the presence of NSP16. Notably, *IFIT2* knockdown had only a modest, nonsignificant impact on replication, contrasting with results obtained with SARS-CoV. Overall, the data indicate that dNSP16 mutant MERS-CoV attenuation is driven by sensitivity to type I IFN mediated by the activity of *IFIT1* rather than augmented IFN responses.

### NSP16 mutant vaccination protects from a lethal MERS-CoV challenge.

**FIG 5:**
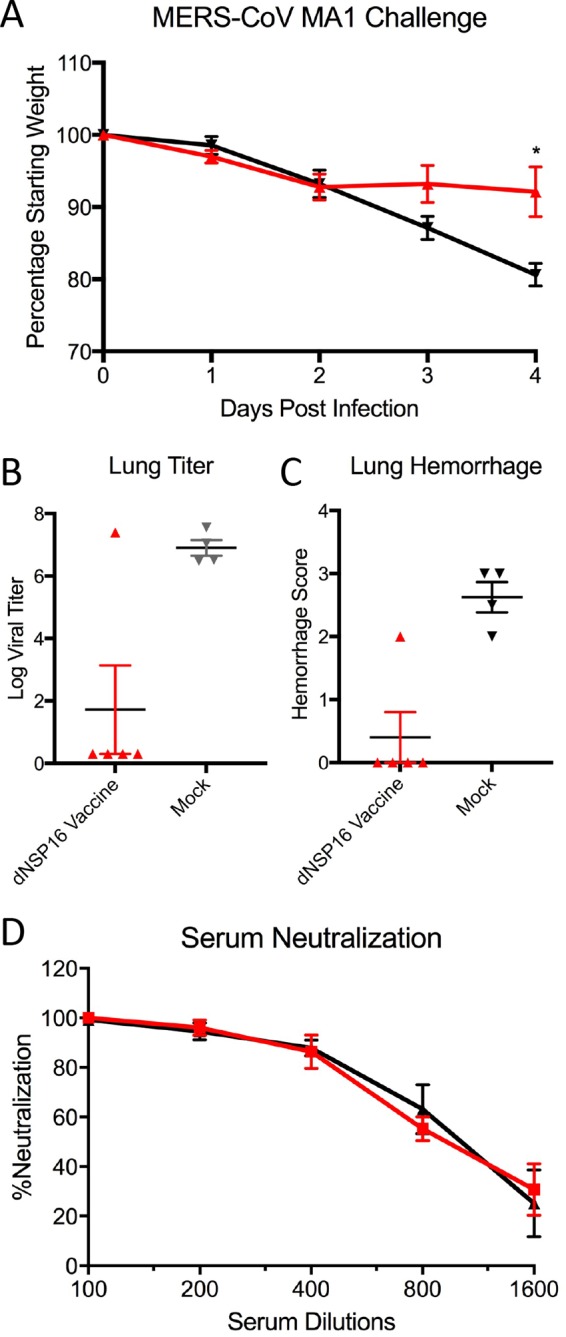
dNSP16 mutant MERS-CoV protects from a lethal challenge. Graphs show weight losses (A), day 4 viral titers (B), and hemorrhage scores (C) following a challenge of 288-330^+/+^ CRISPR-Cas9-targeted mice vaccinated with dNSP16 mutant MERS-CoV (red) or mock vaccinated (black) with 10^6^ PFU of passaged mouse-adapted MERS-CoV (15). (D) Plaque reduction neutralization with serum from WT (black) or dNSP16 mutant (red) MERS-CoV-vaccinated mice. *, *P* < 0.05; **, *P* < 0.01; ***, *P* < 0.001 (Student *t* test). *n* = ≥4 for each experimental group at each time point in multiple experiments.

On the basis of IFN and IFIT1 attenuation phenotypes, targeting of NSP16 offers a potential platform strategy for live attenuated vaccine generation. While previous work by our group had shown that the dNSP16 mutant SARS-CoV conferred protection from a lethal challenge, similar phenotypes in other more distant CoVs are essential for establishing universal principles of attenuation across a virus family. To test this hypothesis, *Dpp4* 288-330^+/+^ mutant mice were vaccinated with dNSP16 mutant MERS-CoV and subsequently challenged with a mouse-adapted MERS-CoV strain (Fig. 5) (15). Following the challenge, dNSP16 mutant-vaccinated mice showed only modest weight loss, in significant contrast to the severe disease seen in the control group (Fig. 5A). In addition, both viral replication and lung hemorrhage were significantly reduced in the context of the dNSP16 mutant vaccine (Fig. 5B and C). Importantly, serum analysis revealed robust virus neutralization with values similar to those seen in serum from WT virus-infected mice (Fig. 5D). Together, the results indicate that dNSP16 mutant MERS-CoV can function as a vaccine platform that not only induces high levels of neutralizing antibodies but provides compete protection from a lethal MERS-CoV challenge.

### NSP16 mutation attenuates mouse-adapted MERS-CoV.

**FIG 6:**
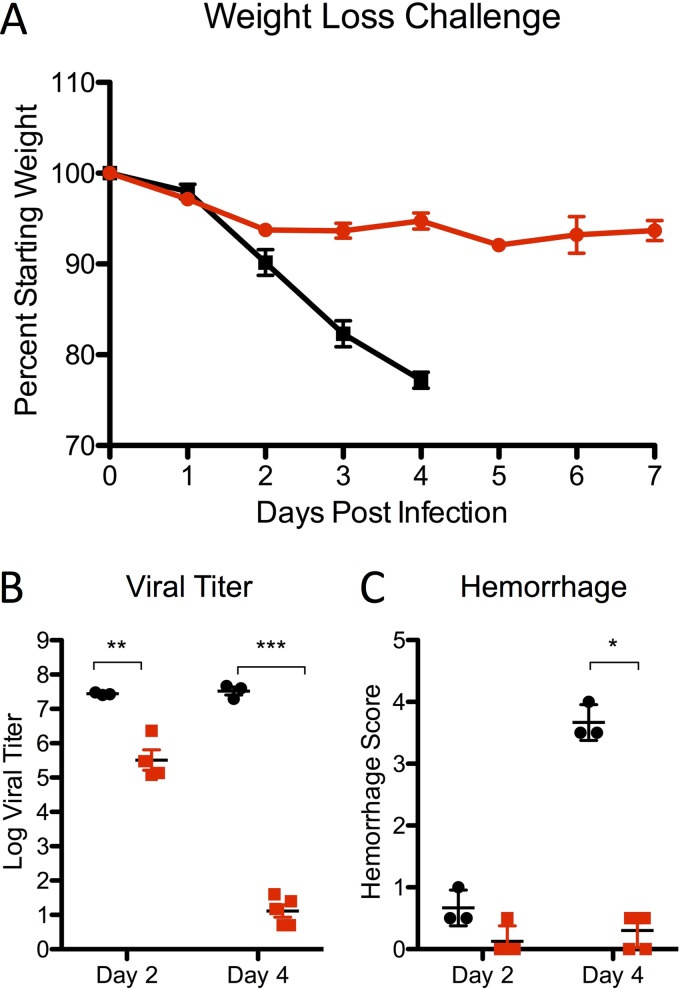
dNSP16 mutation attenuates the virulence of a mouse-adapted MERS-CoV strain. Graphs show weight losses (A), day 4 viral titers (B), and hemorrhage scores (C) following infection of 288-330^+/+^ CRISPR-Cas9-targeted mice infected with 10^6^ PFU of MERS-CoV MA1 (black) or dNSP16 MA1 (red) at days 2 and 4. *, *P* < 0.05; **, *P* < 0.01; ***, *P* < 0.001 (Student *t* test). *n* = ≥3 for each group at each time point in two experiments.

Despite conferring protection in the WT MERS-CoV backbone, it was unclear if the NSP16 mutant would be sufficiently attenuated in a virulent MERS-CoV backbone. To address this question, we inserted the dNSP16 mutation (D130A) into the mouse-adapted MERS-CoV backbone (15). Following infection, mouse-adapted MERS-CoV produced rapid weight loss and death (Fig. 6A). In contrast, the mouse-adapted dNSP16 mutant produced only modest weight loss and 100% survival following infection. In addition, the replication of the dNSP16 mutant was significantly attenuated relative to that of the WT at days 2 and 4 postinfection (Fig. 6B). Finally, hemorrhage scoring of the lung revealed minimal disease in dNSP16 mutant-immunized mice relative to control mice at day 4 postinfection (Fig. 6C). Overall, the results demonstrate robust attenuation of MERS-CoV pathogenesis in the context an NSP16 mutation.

## DISCUSSION

In the context of the ongoing MERS-CoV outbreak, the development of universal platform strategies to attenuate emerging and contemporary CoVs is a significant priority. In this study, we demonstrate the critical importance of NSP16 function to MERS-CoV pathogenesis *in vitro* and *in vivo*. Similar to MHV and SARS-CoV (9, 10), the disruption of 2′*O*-MTase activity in MERS-CoV had no significant impact on replication or early host response patterns *in vitro*. However, dNSP16 mutant MERS-CoV demonstrated attenuated replication and growth in primary HAE cell cultures, as well as reduced disease *in vivo*, relative to the WT virus. Further examination revealed increased sensitivity to type I IFN in an IFIT-dependent manner. Notably, this IFN/IFIT-based attenuation phenotype provided robust protection from a lethal challenge following vaccination with the dNSP16 mutant. Importantly, the NSP16 mutant also was fully attenuated in a highly virulent mouse-adapted MERS-CoV backbone. These results, coupled with previous work with other CoVs, highlight the viral 2′*O*-MTase activity as a potential universal platform for therapeutic treatment and vaccine development for current and future emergent human and animal CoVs.

Similar to previously viral methyltransferase mutants of both flaviviruses and CoVs, substitution with the highly conserved KDKE motif alters the pathogenicity of MERS-CoV. Like SARS-CoV mutants (10), dNSP16 mutant MERS-CoV had normal replication but was attenuated in the context of type I IFN and IFIT activity. Notably, the absence of 2′*O*-MTase activity did not result in an increased type I IFN response, suggesting that loss of NSP16 produces a sensitivity to IFIT activity not seen in the WT virus. While the mutant RNA should induce greater IFN activation through MDA5, multiple other CoV proteins have been shown to disrupt immune recognition (16). Similarly, viral processes including replication within double membrane vesicles may also limit IFN stimulation. Together, the results indicate that even without NSP16, CoVs are able to control IFN stimulation and replicate as efficiently as the WT virus. However, NSP16 is needed to protect the viruses once the IFN-stimulated gene (ISG) effector response is initiated.

The attenuation of the MERS-CoV NSP16 mutant also provides evidence supporting the targeting of viral 2′*O*-MTase as a global therapeutic strategy against emergent RNA viruses. Previous work with both flaviviruses and CoVs has demonstrated robust attenuation by targeting the conserved KDKE motif of the viral 2′*O*-MTase (8, 17, 18). Importantly, 2′*O*-MTase activity is the only known function for NSP16 and it appears completely dispensable for CoV replication in the absence of a strong IFN response (8). In contrast, flaviviruses without *nsp5*-encoded methyltransferase activity are much more sensitive to innate immune responses (19); mutants of West Nile virus, dengue virus, and Japanese encephalitis virus are highly replication attenuated at early time points (20, 21). While diminished virus yields likely reduce flavivirus vaccine utility *in vivo*, robust propagation of both MERS-CoV and SARS-CoV dNSP16 mutants provides a useful parameter for vaccine generation. For both viral families, drugs targeting 2′*O*-MTase activity may have great efficacy if paired with stimulation of the IFN-responsive genes, including that for IFIT1 (8, 17). Overall, the results indicate that viral 2′*O*-MTase activity is a critical determinant of pathogenesis and can be leveraged to develop therapeutic treatments.

NSP16 also represents the third highly conserved CoV protein targeted as the basis of a vaccine platform. Previous work by our group demonstrated the importance of CoV fidelity for infection and pathogenesis (22); disruption of the exonuclease activity of NSP14 rendered attenuated, reversion-proof versions of both MHV and SARS-CoV (22–24). For SARS-CoV, the disruption served as the basis of a successful live attenuated vaccine (22). Similarly, groups have targeted the envelope protein of SARS-CoV and defined a key inflammatory role for E protein in pathogenesis. SARS mutants targeting E function also conferred protection from a lethal challenge (25, 26). For both NSP14 and E mutants, fidelity and inflammation induction are partially responsible for attenuation. However, both viral mutants also maintain replication attenuated in terms of kinetics and yields, potentially complicating their use as vaccine platforms, especially in outbred populations (22, 26). Importantly, the NSP14 mutant has not yet been recovered within the context of MERS-CoV and failure has been reported in group I CoVs (27). Similarly, disruption of the E protein renders MERS-CoV, transmissible gastroenteritis virus, and MHV replication deficient without exogenous complementation (28–30); this broad attenuation of the delta E mutant potentially limits its use as a live attenuated vaccine. In contrast, dNSP16 mutant MERS-CoV maintains robust propagation, as well as IFN/IFIT-based attenuation phenotypes. Together, these results suggest that targeting ofNSP16 may be the most broadly applicable platform for CoV attenuation.

Despite the success of NSP16 mutants in protection studies with SARS-CoV and MERS-CoV, several additional parameters must be considered in the context of CoV vaccination. Previous work by our group has demonstrated failures of CoV subunit vaccines in aged animals and in the context of a heterologous challenge (31). With the aged representing a population with high mortality rates and marginal vaccination success, the efficacy of the NSP16 mutant in this population is paramount in its pursuit as a platform. Similarly, the existence of numerous CoVs in animal populations raises concerns about heterologous challenges from emergent viruses (32, 33). Prior reports had also demonstrated vaccine-induced disease following heterologous challenges with related SARS-like viruses (31, 34). With this in mind, NSP16-vaccinated mice will need to be examined in the context of a heterologous challenge to determine if vaccine-induced pathology occurs. Together, these two factors represent important checkpoints in the pursuit of NSP16 as a universal CoV vaccine platform.

In addition to aging and heterologous challenges, reversion and baseline pathogenesis also represent important risks that must be evaluated in the context of an NSP16 vaccine. While the NSP14 SARS vaccine was absent sterilizing immunity in immunodeficient mice, the lack of reversion over time *in vivo* indicates safety in the approach (22). In contrast, passage of the E mutant rendered a novel mutant that transplanted a critical ion channel function from another viral protein and thus restored partial virulence (35). Studies examining the reversion potential of NSP16 are critical prior to its use as a vaccine; these concerns are especially important considering that both the SARS-CoV and MERS-CoV dNSP16 mutants have robust replication, permitting additional opportunities for reversion (10). In addition, equivalent host immune responses during early infection may produce substantial damage prior to IFN/IFIT-based attenuations, most notably in aged and immunocompromised mice. Therefore, despite the promise of successful attenuation in multiple CoV backbones, several additional metrics must be examined prior to the use of NSP16 mutants as a universal CoV vaccine platform.

Overall, the present study demonstrates that targeting of 2′*O*-MTase activity is a robust strategy to attenuate MERS-CoV and other emergent CoVs. In the absence of NSP16 activity, the MERS-CoV mutant is sensitive to type I IFN in an IFIT-dependent manner, providing a clear attenuation mechanism. Importantly, unlike other conserved CoV platforms, the NSP16 mutant is both viable and robust enough to be used as an effective live attenuated vaccine. While further vaccine characterization is required, the results indicate that disruption of CoV NSP16 activity can be the basis of therapeutic strategies for both current and future emergent CoV infections in both human and animal populations.

## MATERIALS AND METHODS

### Cells and viruses.

The WT, mutant, and mouse-adapted versions of MERS-CoV used in this study were previously described (13, 15) and were cultured on Vero 81 cells grown in Dulbecco’s modified Eagle’s medium or Opti-MEM (Gibco, Carlsbad, CA) and 5% fetal bovine serum (HyClone, South Logan, UT) along with antibiotic/antimycotic (Gibco, Carlsbad, CA). Growth curves in Vero, Calu-3 2B4, and HAE cells were performed as previously described, with examination of multiple samples (*n* ≥3) at each time point (9, 31). Briefly, cells were washed with phosphate-buffered saline (PBS) and inoculated with virus or mock diluted in PBS for 40 min at 37°C. Following inoculation, cells were washed three times and fresh medium was added to signify time zero. Samples were harvested at the time points described. For IFN pretreatments, 100 U/ml recombinant human IFN-β (PBL Laboratories) was added to cells 16 h prior to inoculation, and the cells were infected as described above. Stable shRNA knockdown Vero cell lines for both IFIT1 and IFIT2 were previously described for previous CoV studies and had phenotypic validation (10). All virus cultivation was performed in a biosafety level 3 laboratory with redundant fans in biosafety cabinets as described previously by our group (32, 33). All personnel wore powdered air purifying respirators (3M Breathe Easy) with Tyvek suits, aprons, and booties and were double gloved.

### Construction of WT and NSP16 mutant viruses.

Both WT and mutant viruses were derived from either MERS-CoV EMC or a corresponding mouse-adapted (MA1) infectious clone as previously described (13). For NSP16 mutant construction, the D130A mutation changed the sequence from the MERS-CoV E fragment, which was cloned into the pSMART vector (Lucigen) and used for alanine scanning mutagenesis of conserved residues in nsp16. For the D130A change, a product was generated by PCR with primers against MERS-CoV NSP16 [fragment 1, EMC:E#2(+) (TGAACTACCTGTAGCTGTAG) and EMC:EmuC(−) (NNNNNN**GCTCTTC**TCGCGGAAATAACAAGATCCACTTG); fragment 2, EMC:EmuC(+) (NNNNNN**GCTCTTC**CGCGATGTATGATCCTACTACTAAG) and EMC:E#6(−) (CAACCTCAATACAAGCAGAC)]. The two resulting products were digested with SapI (in boldface) and ligated overnight with T4 DNA ligase. This product was then digested with PpuMI and NsiI and used to replace the region of the EMC E plasmid (puc57) that had been similarly digested. Thereafter, plasmids containing WT and mutant MERS-CoV genome fragments were amplified, excised, ligated, and purified. *In vitro* transcription reactions were then performed to synthesize full-length genomic RNA, which was transfected into Vero E6 cells. The medium from transfected cells was harvested and used as seed stock for subsequent experiments. Viral mutants were confirmed by sequence analysis prior to use. Synthetic construction of NSP16 mutants was approved by the University of North Carolina Institutional Biosafety Committee.

### RNA isolation, microarray processing, and identification of DE.

RNA isolation and microarray processing, quality control, and normalization from Calu-3 2B4 cells were carried out as previously described (36). Differential expression (DE) was determined by comparing virus-infected replicates with time-matched mock-treated replicates. Criteria for DE in determining the consensus ISG list were an absolute log_2_ change of >1.5-fold and a false-discovery rate-adjusted *P* value of <0.05 for a given time point.

### Clustering and functional enrichment.

Genes identified as differentially expressed were used to generate clustered expression heat maps. Hierarchical clustering (using Euclidean distance and complete linkage clustering) was used to cluster gene expression according to behavior across experimental conditions. The David online resource (https://david.ncifcrf.gov/) was used to acquire functional enrichment results for the genes in each cluster. David output was manually summarized for each cluster. Plots were generated with R.

### Ethics statement.

This study was carried out in accordance with the recommendations for the care and use of laboratory animals of the Office of Laboratory Animal Welfare (OLAW), National Institutes of Health. The Institutional Animal Care and Use Committee (IACUC) of the University of North Carolina at Chapel Hill (UNC; permit A-3410-01) approved the animal study protocols used in this study (IACUC protocols 15-009 and 13-072).

### Mouse infections and vaccinations.

Ten- to 20-week-old BALB/c (Envigo/Harlan) or CRISPR-Cas9-targeted 288-330^+/+^ C57BL/6 mice were anesthetized with ketamine and xylazine (in accordance with UNC IACUC guidelines) and intranasally inoculated with a 50-µl volume containing 10^6^ PFU of WT MERS-CoV, dNSP16 mutant MERS-CoV, mouse-adapted variants, or PBS (mock inoculation) as indicated in the figure legends. Infected animals were monitored for weight loss, morbidity, and clinical signs of disease, and lung virus titers were determined as described previously (37). *In vivo* adenovirus transduction with *Dpp4* were performed as previously described (14). For vaccination experiments, 10- to 20-week-old 288-330^+/+^ mice were infected with 10^6^ PFU of dNSP16 mutant MERS-CoV as described above, monitored for clinical symptoms for 7 days, and then challenged 4 weeks postvaccination with 10^6^ PFU of mouse-passaged MERS-CoV MA1. Animal housing, care, and experimental protocols were in accordance with UNC IACUC guidelines.

### Data availability.

Raw microarray data for these studies were deposited in publicly available databases at the National Center for Biotechnology Information (NCBI) Gene Expression Omnibus (37) and are accessible through GEO series GSE65574.
